# Mechanochemical Synthesis of Rosin-Modified Montmorillonite: A Breakthrough Approach to the Next Generation of OMMT/Rubber Nanocomposites

**DOI:** 10.3390/nano11081974

**Published:** 2021-07-31

**Authors:** Elaheh Esmaeili, Seyyed Amin Rounaghi, Jürgen Eckert

**Affiliations:** 1 Department of Chemical Engineering, Birjand University of Technology, Birjand, Iran; 2 Research and Development Laboratory, Nano Parmin Khavaran Company, Birjand, Iran; rounaghi@birjandut.ac.ir; 3 Erich Schmid Institute of Materials Science, Austrian Academy of Sciences, Jahnstraße 12, A-8700 Leoben, Austria; 4 Department of Materials Science, Chair of Materials Physics, Montanuniversität Leoben, Jahnstraße 12, A-8700 Leoben, Austria

**Keywords:** mechanochemistry, rubber-compatible organo-montmorillonite, gas permeation, inner liner, nanocomposite

## Abstract

The current investigation presents a green mechanochemical procedure for the synthesis of a special kind of rubber-compatible organo-montmorillonite (OMMT) for use in the inner liner compound of tires. The compatibility character of the OMMT arises from the mechanochemical reaction of the raw bentonite mineral and gum rosin as some of the organic constituents of the inner liner composition. The monitoring of OMMT synthesis by various characterization techniques reveals that gum rosin gradually intercalates into the montmorillonite (MMT) galleries during milling and increases the interlayer spacing to 41.1 ± 0.5 Å. The findings confirm the simultaneous formation of single- or few-layered OMMT platelets with average sizes from the sub-micron range up to several micrometers during the milling process. The mechanical properties of the OMMT/rubber nanocomposite, such as tensile strength, tear resistance and elongation, present a good enhancement in comparison to the un-modified material. Moreover, the organo-modification of the inner liner composition also leads to a property improvement of about 50%.

## 1. Introduction

Bentonite is a swelling aluminum phyllosilicate clay which has attracted tremendous attention because of the layered structure of the montmorillonite (MMT). Besides its low price, the aluminosilicate-layered backbone of montmorillonite makes bentonite a very promising natural candidate for many applications, such as oil-field scale inhibition, pollutant removal, wastewater treatment, rheology control of suspensions, catalysis, composites, packaging, etc. [1,2,3,4,5,6].

However, due to the inorganic nature of bentonite, it is not compatible with organic media and, hence, requires being organo-modified [7,8,9,10]. Many different methods, including ion exchange [11,12], grafting of organic compounds [13,14], acid–base reactions [15], pillaring by different types of polycations [16,17], ion-dipole interaction [11,18], etc., were applied to modify the bentonite minerals. Among these, the conventional wet cation exchange technique has been commercialized and widely used for the synthesis of organo-montmorillonite (OMMT). However, some drawbacks regarding the environmental and economic aspects have remained challenging. First of all, the use of intercalating agents in wet chemical methods is limited to water-soluble organic compounds, such as alkyl quaternary ammonium compounds, which are environmentally hazardous [19,20]. Moreover, the cation exchange reaction most often is not fully completed. Therefore, organic waste remains, which not only increases the production costs, but also causes environmental pollution. This technique also requires a heating step at temperatures ranging from 50 °C up to 80 °C, such that lower temperatures result in a lower cation exchange efficiency [21]. The large amount of water and energy consumed during several filtrating, drying and grinding steps is another issue limiting wet chemical-based methods [21,22]. Considering these facts, a versatile approach for OMMT production is strongly desirable to address the issues arising from common wet chemical methods.

During the two recent decades, mechanochemical approaches have attracted increasing attention due to the solvent-free, cost effective and environmentally friendly synthesis of a wide variety of nanomaterials [23,24,25,26]. Mechanochemical synthesis through conventional ball milling is the most prevalent route in which chemical or physical transformations are triggered by the energy of ball impacts [27,28,29]. Mechanochemical synthesis of OMMT was first reported by Ogawa et al. through a solid-state ion exchange reaction between montmorillonite and alkylammonium salts [30,31]. In this case, OMMT is synthesized by the simple grinding of powder mixtures in an agate mortar at room temperature. In spite of the ease of using organoammonium salts, a post-washing process is still required for the removal of the metal halide by-product. Solid-state intercalation of polar organic molecules into the montmorillonite interlayers through an ion-dipole mechanism is another synthesis route, where the polar group of the organic molecule interacts with interlayer cations [7,32]. According to this approach, the subsequent washing process is avoided due to the lack of formation of solid by-products. Furthermore, a wide spectrum of organic molecules, such as amines, amides, amino acids, carboxylic acids, etc., can be feasibly intercalated into the MMT gallery to form OMMT [31,33,34]. On the other hand, non-ionic surfactants don’t change the charge density of the platy clays; therefore, they cause an increased hydrophobicity of the modified clays and unique improvements compared to the ionic ones [32].

Commercial OMMTs are globally confined to alkylammonium-modified MMTs. On one side, these kinds of OMMTs are potentially toxic, and on the other side, they exhibit a limited compatibility with some polymers [35,36,37,38,39]. Hence, the development of a new class of OMMTs which are safe and fully adapted with the base polymer matrix is highly demanded and of great importance. The excellent compatibility of organic constituents of the unmodified polymer phase makes them promising candidates as intercalating agents for the preparation of polymer-compatible OMMT. However, the organic nature of these constituents and their low solubility in aqueous media hinder their application as an intercalating agent by wet chemical methods. To overcome this problem, the ion-dipole hypothesis is applied to open a window on using new classes of organic materials in the solid phase for the modification of MMT with uncommon intercalating agents which are not normally used as intercalating agents through conventional wet chemical methods.

Gum rosin is one of those substances widely used in polymers, especially in the tire industry as an adhesion promoter [40]. This non-ionic solid resinous substance is naturally composed of around 90% resin acids, whose 90% are isomeric with abietic acid (C_20_H_30_O_2_), containing conjugated double bonds and a single carboxylic acid. The other residual percentage (about 10%) includes neutral matters with non-conjugated double bonds [40,41,42]. The eco-friendly, green and organic nature of gum rosin as a tire ingredient makes it a very promising candidate for the production of a rubber-compatible OMMT.

In the present investigation, the objective is to organically modify bentonite powder with gum rosin as intercalating agent through a solvent-free mechanochemical approach. The organo-modified MMT is evaluated for its use in the inner liner composition of tires through investigating the dispersion behavior and the exfoliation of platy MMTs within the rubber composition. Moreover, physical and mechanical properties as well as the gas permeability of the OMMT-modified inner liner composite are also studied.

## 2. Materials and Methods

### 2.1. Materials

**Scheme 1:**
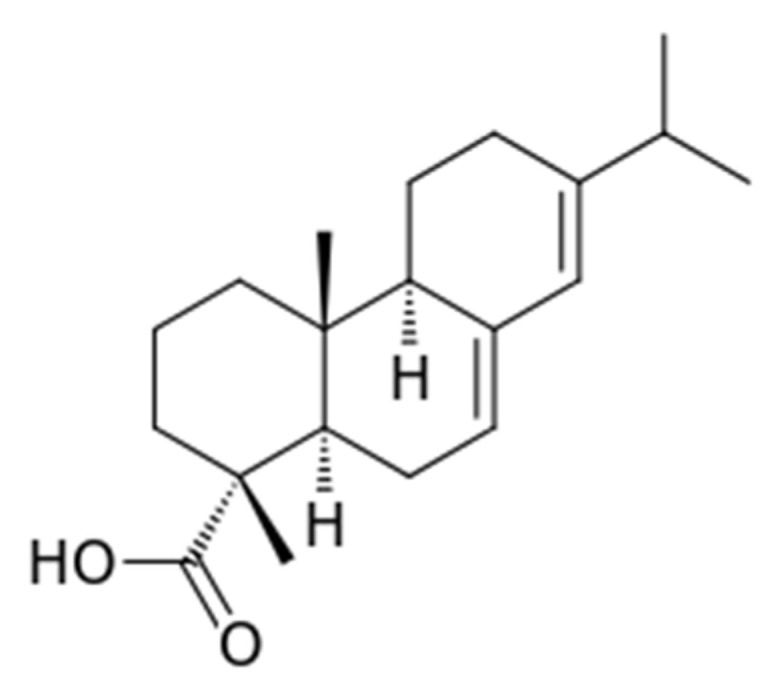
Chemical structure of abietic acid.

All the chemicals and minerals were used as-received without further treatment. Bentonite with a cation exchange capacity (CEC) of 103 meq/100 g was obtained from the bentonite mines of the South Khorasan province of Iran and supplied by Vivan Industrial Group. Gum rosin WW grade was purchased from Sadkem (London, UK). The chemical structure of abietic acid as the main constituent of gum rosin is illustrated in Scheme 1.

### 2.2. OMMT Synthesis

A solid-state mechanochemical method was applied to synthesize the OMMT. The reaction mixture was obtained by the addition of 4.1078 g of bentonite powder and 1.9195 g of gum rosin in accordance with the cation exchange capacity of raw bentonite and milled using a planetary ball mill for 4 h at a rotational speed of 250 rpm. The weight ratio of the 10 mm hardened steel balls to the reaction mixture was adjusted to be 30:1.

### 2.3. Preparation of OMMT/Rubber Nanocomposites

The OMMT/rubber nanocomposites were prepared by the addition of 4 phr and 7 phr synthesized OMMT to the desired amounts of natural and synthetic rubbers, fillers, tackifiers, softeners and antioxidants, as demonstrated in Table 1. To enhance the processability of the compound, parts of carbon black as filler were substituted by the same amounts of OMMT. To produce the master batch, all the constituents were mixed by an internal banbury mixer (Pomini MIX32) (Pomini Rubber & Plastics Srl, Milan, Italy) to obtain a homogeneous composition. Then, the resulting master batch was allowed to relax for 24 h.

The final inner liner composition of a tire was produced by the addition of appropriate amounts of vulcanizing agents to the master batch on a two-roll mill (Battaggion, Bergamo, Italy) for 10 min. The vulcanizing agents consisted of sulfur, MBTS, CBS, vultac and zinc oxide. The resulting final composition was cured in suitable molds at 145 °C under 110 bar pressure for 30 min.

In order to investigate the dispersion behavior of the MMT platelets within the rubber mixture, 4 phr of the synthesized OMMT was added to the rubber mixture including all the natural and synthetic rubbers on a laboratory two-roll mill for 20 min. In fact, the other constituents of the master batch inner liner compound were neglected.

### 2.4. Materials Characterization

X-ray diffraction (XRD) analyses were conducted by means of a X’Pert PRO MPD diffractometer (PANanalytical B.V., Almelo, Netherlands) with Cu Kα radiation (λ = 1.54060 Å) to determine the phase evolution, intercalation and exfoliation of OMMT during the production process. The chemical structure of the prepared OMMT was characterized using Fourier transform infrared spectroscopy (FTIR) via the KBr pellet technique. The concentration of the sample in the KBr was kept at 0.2 wt.%. Data were recorded with a ThermoNicolet Avatar 370 spectrometer (Nicolent, Maison, Madison, WI, USA) in the optical range of 400–4000 cm^−1^ at a resolution of 4 cm^−1^. Transmission electron microscopy (TEM) images were obtained using a Philips CM300 instrument (Philips Electronics, Eindhoven, Netherlands) operated with an accelerating voltage of 150 kV to study the extent of intercalation or possible exfoliation of the OMMT. The powdered TEM samples were prepared by suspension of OMMT particles in ethanol followed by sonication in a low-energy ultrasonic bath for few minutes, and then putting a droplet of suspension on a carbon-coated copper grid. For the rubber samples, thin sections (less than 100 nm) were prepared using a cryo-ultramicrotome (Leica EM UC7) (Leica Microsystems Inc., Buffalo Grove, IL, USA) and the sections were supported onto a copper grid for the subsequent analysis. The morphology of the powder was also investigated with a Hitachi S4160 field emission scanning electron microscope (FESEM) (Hitachi Instrument, Tokyo, Japan), operated at 20 kV. The samples were gold-coated with a thickness of less than 10 nm prior to the analysis. Atomic force microscopy (AFM) images were recorded in air and ambient conditions using a NT-MDT AFM apparatus (NT-MDT Spectrum Instruments, Moscow, Russia) in non-contact mode with a NSG10 tip (TipsNano Co., Tallinn, Stonia) at 190 KHz resonance frequency and a scanning rate of 0.3 Hz. For AFM sample preparation, a very small quantity of powder was sonicated in ethanol for 10 min, followed by drop casting on a silicon wafer substrate. The measurements were conducted after solvent evaporation and data were collected over 50 particles of three similarly prepared samples. The AFM images were processed by means of the Nova Px 3.1.0 (NT-MDT Spectrum Instruments, Moscow, Russia) to obtain the numerical data. For thermal gravimetric analysis (TGA), 7.723 and 7.295 mg of gum rosin and the synthesized OMMT were respectively weighed and located at the platinum pans. The measurements were conducted on a TGA-50 Shimadzu thermogravimetric analyzer (Shimadzu Corporation, Kyoto, Japan) using a heating rate of 10 °C/min under air atmosphere.

The physical and mechanical properties of the resulting rubber and OMMT/rubber nanocomposites, such as tensile strength, elongation at break (%), modulus 300% and tear resistance, were characterized by a Dynamometer Hounsfield H10KS tensile (Hounsfield Test Equipment Ltd., London, UK) testing instrument. In order to obtain reliable results, the average of three samples is reported. The thickness of the samples was tested by the use of a digital thickness tester (Dongguan Liyi Environmental Technology Co., Ltd., Guangdong, China). The average of at least ten readings was taken for further calculations. Hardness measurements were also performed using a Zwick 3100 model (ZwickRoell, Ulm, Germany). The vulcanization characteristics of the compounds were checked with an Alpha Technologies MV2000E Mooney viscometer (Alpha Technologies, Hudson, NY, USA). The air permeability (OTR) of the samples was measured in a permeation cell consisting of two compartments separated by the molded samples. Following the evacuation of the chamber, 2 bar gas pressure was introduced into the upstream compartment of the cell. The variation of the gas pressure in the downstream compartment was recorded. Finally, the permeability coefficient was calculated in Barrer through the equation (1 Barrer = 1 × 10^−10^ cm^3^ (STP) cm/cm^2^ s cmHg), as reported previously [43,44]. Data were taken from the average of two measurements.

## 3. Results and Discussion

**Figure 1:**
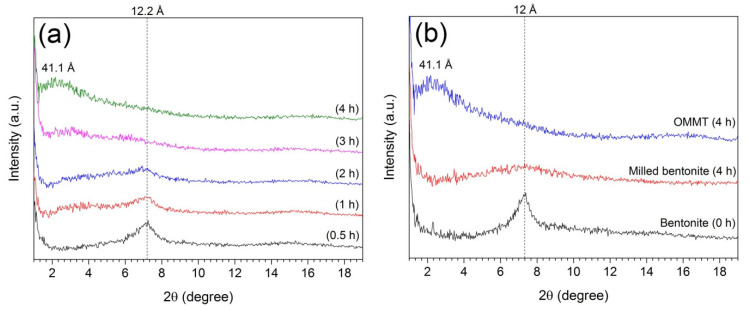
X-ray diffraction patterns of (**a**) the bentonite/gum rosin powder mixture at various milling times and (**b**) the OMMT compared to the pristine and milled bentonite.

Figure 1a depicts the XRD patterns of the pristine bentonite and the gum rosin mixture after various milling times. The strong and broad peak observed at the diffraction angle (2θ) of around 7.2° for the 0.5 h-milled sample corresponds to silicate clay galleries with an average basal spacing of 12.2 ± 0.5 Å. This peak is slightly shifted to lower angles in comparison with the pristine bentonite (Figure 1b), which may be due to the intercalation of gum rosin into the MMT interlayers at the first stage of milling. Based on Figure 1a, the intensity of the mentioned peak slightly decreases with increasing milling time. Meanwhile, another broad peak gradually appears at diffraction angles of 2–4°. In other words, the milling process plays a significant role in the gradual intercalation of the gum rosin into the MMT galleries. According to Figure 1a, the platelet basal spacing increases from 12.2 ± 0.5 Å up to 41.1 ± 0.5 Å when the bentonite powder and gum rosin are milled for 0.5 up to 4 h, respectively.

Figure 1b compares the effect of milling on the clay XRD pattern in the absence or presence of the intercalating agent at the same milling time (4 h). For better judgment, the corresponding pattern of pristine bentonite is also provided. As shown, the peak intensity at 2θ = 7.4° associated with the reflection of the (001) planes of montmorillonite in the pristine bentonite (unmilled) sample is drastically decreased when the bentonite powder is milled for 4 h in the absence of gum rosin. It is worth noting that the milling process has no considerable effect on the destruction of the MMT structure. Indeed, the decrease in the intensity of the basal reflections can be mainly attributed to distortion, delamination and pilling off the MMT layers [45].

According to Figure 1b, the milling of the bentonite mineral in the presence of gum rosin leads to the appearance of a peak at 2.2° as a result of the intercalation of organic molecules into the MMT interlayers. This result corroborates the synergic effect of milling and gum rosin as the intercalating agent for the synthesis of partially exfoliated organo-modified MMT.

**Figure 2:**
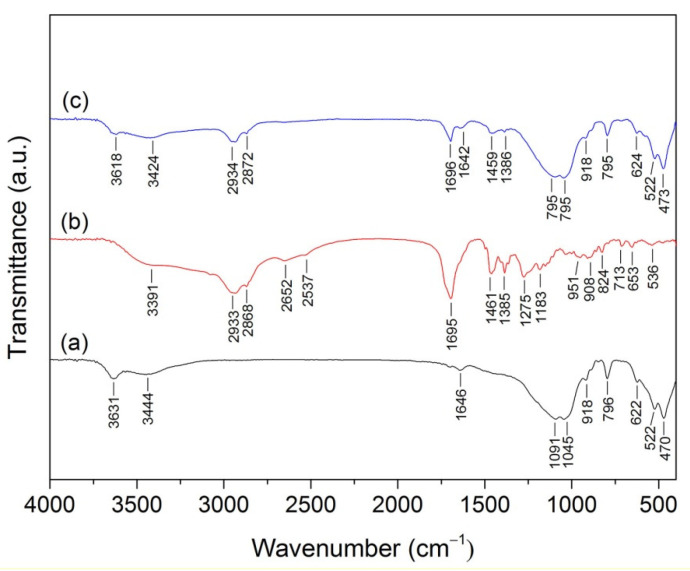
FTIR spectra of (**a**) bentonite, (**b**) gum rosin and (**c**) OMMT.

The chemical structures of the MMT, gum rosin and the synthesized OMMT can be elucidated from the corresponding FTIR spectra in Figure 2. As shown, the FTIR spectrum of the pristine bentonite (Figure 2a) is in good accordance with the representative characteristics of the MMT phase. The peak at around 3631 cm^−1^ is related to the stretching vibrations of OH bonded with Al^3+^, revealing the presence of the octahedral layers of Al in the MMT structure [46,47,48]. The stretching vibrations of 3444 cm^−1^ are associated with the hydroxyl groups of free and interlayer water molecules [48]. Furthermore, the peaks located at 1646 cm^−1^ are indicative of the bending vibrations of water molecules (H–O–H) on the clay surface. The peaks appearing at around 470 cm^−1^ and 522 cm^−1^ are assigned to the bands related to bending vibrations of Si–O–Si and Al octahedral layers (Si–O–Al), respectively [46,47]. The appearance of the strong peak at 1045 cm^−1^ refers to the stretching vibration modes of Si–O bonds, and the other weak peak centered at 918 cm^−1^ is related to the Al–Al–OH bending vibrations on the edges of the clay mineral [46,48]. The presence of the other peaks at frequencies of 875 cm^−1^ and 836 cm^−1^ is also respectively attributed to the partial substitution of Fe (Al–Fe–OH) and Mg (Al–Mg–OH) in the octahedral layers [48].

The FTIR spectrum of gum rosin in Figure 2b shows a prominent peak at 1695 cm^−1^ arising from C=O stretching bonds of the carboxyl group in abietic acid. Furthermore, the characteristic peaks at 2933 cm^−1^ and 1385 cm^−1^ are attributed to the stretching vibration modes of –OH and –C–O bonds of carboxylic acid, respectively [38,49,50]. The peaks at 2866 cm^−1^ and 2933 cm^−1^ are respectively associated with CH_2_ symmetric and asymmetric stretching vibration modes [51]. The peak in the range of 650–1000 cm^−1^ belongs to the C–H out-of-plane bending, and the one centered at about 1461 cm^−1^ corresponds to CH_2_ bending vibrations [39].

It is obvious that the spectrum associated with the organo-modified MMT (Figure 2c) is a combination of all assignments of pristine bentonite mineral and gum rosin. The small changes in frequency shifts and the decreased intensity of some peaks reveal the formation of some new interactions within the bentonite mineral and gum rosin. For instance, the slight upward peak shift of the CH_2_ bonds to 2934 and 2872 cm^−1^ in the FTIR spectrum of OMMT points to the gauche conformation of organic molecules within the MMT galleries [52].

**Figure 3:**
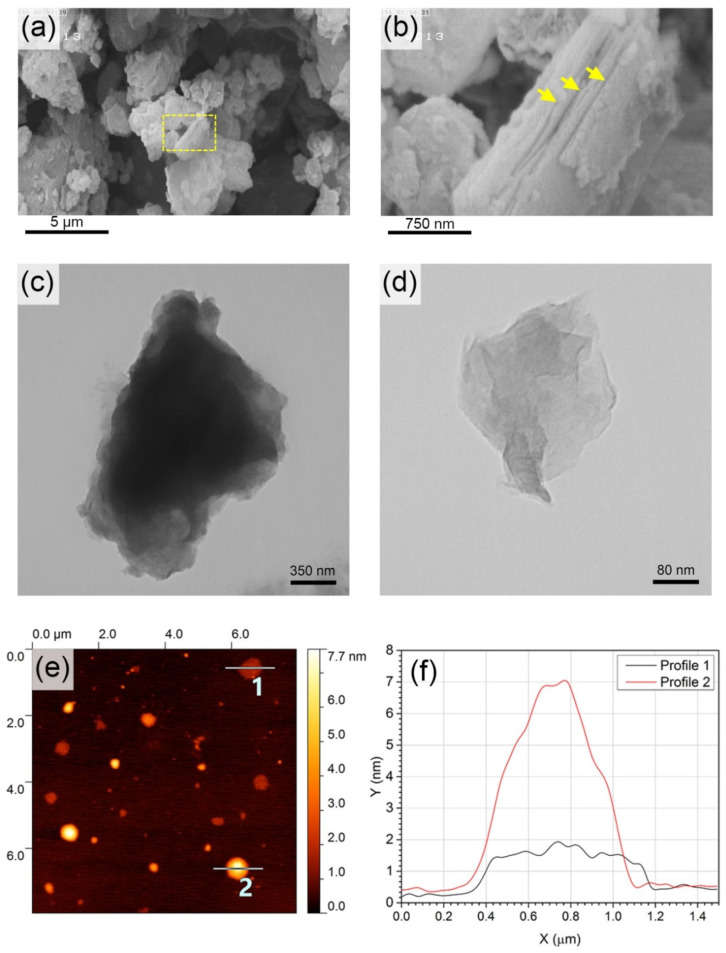
(**a**) FESEM image of the synthesized OMMT powder and (**b**) a higher magnification image from the selected area in (**a**) where arrows denote the lamellar structure of the OMMT particle, TEM micrographs of (**c**) intercalated and (**d**) exfoliated OMMT particles, (**e**) AFM image and (**f**) corresponding line profiles of the synthesized OMMT.

The morphology and structure of the OMMT particles were further investigated by FESEM, TEM and AFM analyses (Figure 3). The FESEM micrograph of the synthesized OMMT (Figure 3a) exhibits irregular shapes of aggregated particles with various sizes, varying from the sub-micron range to several microns. The high magnification FESEM image of a specific particle in Figure 3b reveals the layered structure of OMMT, as indicated by arrows in the figure. TEM observations demonstrate two distinct structures for OMMT particles consisting of relatively thick intercalated layers (Figure 3c) and exfoliated ones with a few nanometers thickness (Figure 3d). On the other side, AFM imaging also confirms the presence of exfoliated MMT platelets with various aspect ratios whose thicknesses are in the nano-sized range (Figure 3e). According to Figure 3f, the thickness values of two typical MMT platelets are measured to be 13 ± 3 Å and 64 ± 3 Å, respectively, assigned to an individual single layer and about five-layered MMT intercalated stacks. Considering the TEM and AFM observations, it is deduced that the current mechanochemical process can simultaneously lead to organic modification and exfoliation of the MMT platelets, which is completely in accordance with the aforementioned XRD results. Therefore, the milling process has a significant effect on the intercalation of the gum rosin into the MMT platelets through weakening the van der Waals interaction within the MMT layers, such that the layers are delaminated by the impacts of the milling balls. It is worth noting that the mechanochemically synthesized OMMT particles have rounded edges with various sizes and thicknesses which differ from those reported in the literature for regular MMT particles [53]. In other words, the mechanical forces induced by ball milling lead to simultaneous intercalation, exfoliation and fracture of MMT platelets. Hence, the size and morphology of the particles strongly depends on which mechanism dominates for each particle.

**Figure 4:**
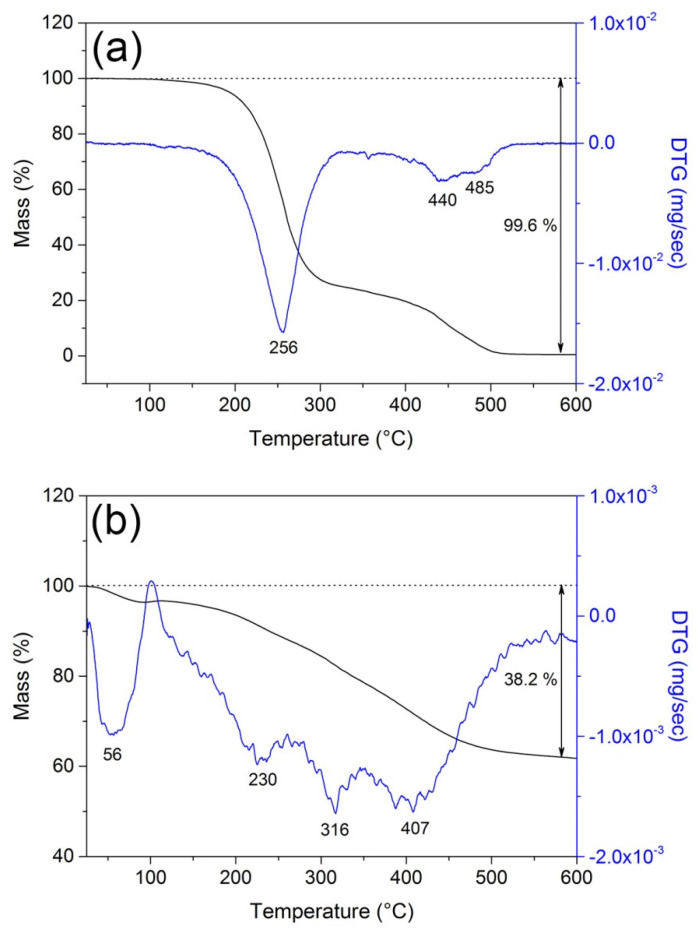
Thermogravimetric analysis results of (**a**) the gum rosin and (**b**) the prepared OMMT.

The thermal stability of gum rosin and synthesized OMMT was studied by thermogravimetric analysis (Figure 4). The gum rosin undergoes an overall mass loss of 99.6 ± 0.1% during oxidation, based on which the residual part is related to the ash content. Moreover, it is deduced from both TGA and DTG curves in Figure 4a that the degradation of gum rosin occurs in two dominant stages. The main stage at the temperature range of 215–276 °C, accompanied by a peak at 256 °C in the corresponding DTG curve, is attributed to the degradation of the abietic acid structure [37,54,55,56]. The second weight loss at 418–495 °C, in line with the peaks in the DTG curve at 440 °C and 485 °C, is probably associated with degradation of abietic acid residues and their oxidation products [57].

Figure 4b illustrates the thermal oxidation behavior of the synthesized OMMT. The weight loss of about 4%, accompanied by the DTG peak at 56 °C, is related to the adsorbed water on the OMMT surface. The low-temperature weight loss of the adsorbed water molecules confirms that the MMT galleries are fully organo-modified by the gum rosin through the mechanochemical reaction. The TGA curve also presents the main weight loss in the range of 150–500 °C, accompanied by three peaks at 230 °C, 316 °C and 407 °C in the DTG curve. Since neat bentonite does not undergo any weight loss in the range of 170–500 °C, these peaks are ascribed to the stage-wise degradation of gum rosin in the MMT galleries [47]. The first stage at 230 °C is attributed to the removal of the surfactants physically adsorbed on the external surface of the MMT [58,59,60]. The second peak at 316 °C is assigned to gum rosin species located at the pores between bentonite particles, such that a house-of-cards structure is formed [59,60]. The last DTG peak centered at about 407 °C is associated with decomposition of the intercalated gum rosin into the MMT interlayers through bonding to surface sites, occurring at higher temperatures than for bulk species, as reported in the literature [60,61].

**Figure 5:**
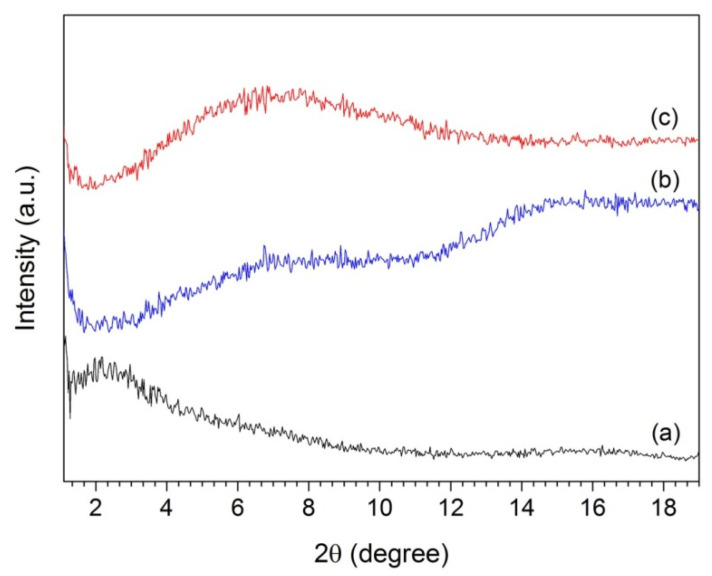
XRD patterns of (**a**) the synthesized OMMT, (**b**) the mixture of rubbers and (**c**) the OMMT-modified rubber.

Figure 5 presents XRD patterns of the OMMT, the mixture of rubbers and the OMMT-modified rubber. As stated previously, the OMMT-modified rubber composition consists of a mixture of rubbers and OMMT to prevent any interference and any diffraction overlaps originating from the other constituents of the inner liner composition during the measurements. As seen, the XRD pattern of the OMMT shows a broad reflection peak related to the (001) basal planes of the MMT. Meanwhile, the XRD patterns of both the rubber matrix and the organo-modified one display a characteristic amorphous background at 2θ angles higher than 4°, arising from the non-crystalline nature of polymer networks. The absence of the (001) basal plane of the MMT in the organo-modified rubber is due to the exfoliation of MMT platelets within the rubber matrix during mixing on the two-roll mill [62,63].

**Figure 6:**
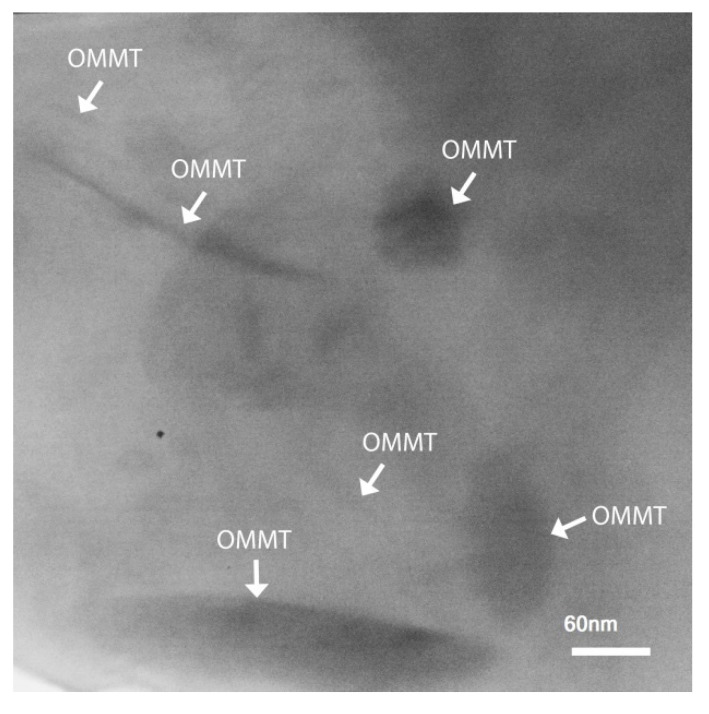
A representative TEM image of the OMMT-modified rubber. The arrows indicate the randomly orientated OMMT layers.

A typical TEM image of the OMMT-modified rubber is shown in Figure 6. Since the OMMT is the only particle-shaped ingredient in the polymeric phase, the darker regions reveal the presence of few-layer OMMT platelets. The existence of exfoliated or single-layer OMMTs can hardly be distinguished from the rubber matrix by the means of TEM measurements due to the limited resolution/contrast, but the presence of exfoliated platelets can be recognized through the AFM analyses of the OMMT powder (Figure 3e,f) and the XRD results of OMMT-modified rubber (Figure 5c). Furthermore, based on Figure 6, the OMMT layers show different sizes and random orientations, as also demonstrated by others [64].

Table 2 summarizes the results of the mechanical properties and air permeation of the unmodified inner liner composition and the OMMT/rubber nanocomposites. According to the literature [65,66,67], the optimum amount of OMMT is found to be in the range of 3–5 phr. However, for a better comparison, the effect of two different parts of 4 and 7 phr of OMMT on the physical and mechanical characteristics of the resulted nanocomposites are investigated. As reported in Table 2, the addition of OMMT to the inner liner composition has a positive impact on some of the mechanical properties. Indeed, the tensile strength, elongation at break and tear resistance are 10.5 ± 0.3 MPa, 537 ± 11 MPa and 36.6 ± 1.6 kN/m, respectively, for the unmodified inner liner composition, whereas values of 11.1 ± 0.2 MPa, 548 ± 10 MPa and 42.3 ± 1.2 kN/m are obtained for the 4 phr OMMT/rubber nanocomposite, respectively. The addition of 7 phr OMMT into the inner liner composition results in the decreased values of 10.76 ± 0.2, 551 ± 8 and 34.1 ± 1.4 for the mentioned properties, correspondingly. These findings show that a lower amount of OMMT is more effective to reinforce the inner liner composition through bridging the rubber molecules with the interface of the OMMT platelets. The higher values of tensile strength and elongation at break for the sample containing a smaller dosage of the organo-modified MMT (4 phr) refers to the formation of dispersed OMMT particles within the matrix, which in turn improves the flexibility of the macromolecules [65,68]. The tear resistance results show a maximum value when the smaller dosage amount of OMMT is applied, whereas the aggregation of OMMT particles creates weak points which promote crack propagation.

The modulus 300% is measured to be 5.4 ± 0.1 MPa for the un-modified material; meanwhile, it reaches values of 4.9 ± 0.1 MPa and 5.68 ± 0.1 for the nanocomposites, respectively modified by 4 phr and 7 phr OMMT. The lower modulus for the smaller amount (4 phr) is attributed to the sliding of the rubber chains within the OMMT galleries. For higher amounts (7 phr), the possible aggregation of OMMT platelets may prevent the slipping of rubber chains and, consequently, enhance the modulus. This hypothesis is supported by the high modulus of carbon black-formulated compounds in which the structural networks of carbon black confine the polymer chains and prevent the slipping of the macromolecules. [65,66].

According to Table 2, there is a descending tendency for the Mooney viscosity of the raw composition of the 4 phr OMMT/rubber nanocomposite when compared to the un-modified sample. This may result from the lubricating effect of the platy OMMTs which causes sliding of the rubber molecules and orienting the stacked OMMTs due to the applied shear [66]. It is obvious that the Mooney viscosity is increased for the 7 phr OMMT/rubber nanocomposite influenced by the formation of OMMT aggregates within the rubber matrix.

The hardness values are essentially unchanged, showing only a negligible decrease (~1%). As known, most of the surfactants utilized for the organo-modification of MMT, such as amines and quaternary alkyl ammonium substances, decrease the scorch time of the elastomeric composites through decreasing the activation energy and accelerating the vulcanization system [69]. Meanwhile, the current synthesized OMMT exhibits no significant effect on the curing process, basically due to the good compatibility of the intercalating agent (gum rosin) with the other constituents of the inner liner compound.

Based on the results of Table 2, the gas permeability strongly decreases for the 4 phr and 7 phr OMMT/rubber nanocomposite, such that the gas barrier capacity is enhanced up to around 150% and 122%, respectively. It is obvious that the barrier properties are significantly improved when the silicate platelets are uniformly dispersed within the rubber matrix [70,71,72,73]. As confirmed by the XRD, AFM and TEM results, the presence of exfoliated silicate clay layers with a proper aspect ratio may be responsible for the tortuous path. In fact, these indirect paths prolong the gas exit time from the inner liner nanocomposite layer and improve the gas barrier properties.

The utilization of an additive of the inner liner composition for the modification of MMT through a facile mechanochemical process gives a promising perspective view for the synthesis of the next generation of OMMTs for further applications in various systems, such as packaging, membranes, films, containers, tires, medicine, etc. This breakthrough approach definitely introduces highly compatible and efficient OMMTs to different industries. Moreover, this concept opens a new window on various layered materials, such as graphene.

## 4. Conclusions

A solid-state mechanochemical technique was applied to prepare a rubber-compatible OMMT for application in the inner liner layer of tires. To reach a good compatibility, the natural bentonite mineral was milled with gum rosin as one of the organic constituents of the inner liner composition. The results confirm the crucial role of milling on the intercalation of the gum rosin into the MMT interlayers, as well as the subsequent exfoliation of MMT platelets. According to TGA analysis, gum rosin is successfully intercalated into the MMT galleries; however, some amount of the surfactant is adsorbed either on the external MMT surface or between the bentonite pores. XRD and TEM of the organo-modified MMT also indicate the presence of intercalated/exfoliated structures of OMMTs within the rubber matrix when compared to the non-modified rubber mixture. This is due to the good compatibility of the OMMT with the inner liner composition. The resulting OMMT/rubber nanocomposite exhibits improved mechanical and barrier properties. The incorporation of 4 phr OMMT into the inner liner composition shows about 50% improvement compared to the unmodified material.

## Figures and Tables

**Table 1:** Material specifications of the base inner liner master batch compound.

Material Type	Phr (Parts Per Hundred Rubber)
Natural and synthetic rubber (including SMR, SBR and chlorobutyl rubber)	100
Carbon black	46.59
Aromatic oil	4.33
Gum rosin	2.63
Calcium carbonate	17.4
Stearic acid	1.13
Magnesium oxide	0.31
Others (Riwax, Anox, …)	0.85

**Table 2:** The effect of OMMT on the physical and mechanical properties and the air permeation of the inner liner composition.

Properties	UnModified Inner Liner Composition	4 Phr OMMT/Rubber Nanocomposite (% Change)	7 Phr OMMT/Rubber Nanocomposite (% Change)
Tensile strength (MPa)	10.5 ± 0.3	11.1 ± 0.2 (+6.1 ± 0.9)	10.76 ± 0.2 (+2.5 ± 1.0)
Elongation at break (MPa)	537 ± 11	584 ± 10 (+9.0 ± 0.6)	551 ± 8 (+2.6 ± 0.6)
Modulus 300% (MPa)	5.4 ± 0.1	4.9 ± 0.1 (−10.2 ± 0.4)	5.6 ± 0.1 (+5.6 ± 0.1)
Tear resistance (kN/m)	36.6 ± 1.6	42.3 ± 1.2 (+16.0 ± 2.0)	34.1 ± 1.4 (−6.8 ± 0.2)
Hardness (Shore A)	57 ± 1	56 ± 1 (−1.75 ± 0.03)	56 ± 1 (−1.75 ± 0.03)
Mooney viscosity (MU)	47.6 ± 0.4	42.8 ± 0.3 (−10.0 ± 1.4)	57.1 ± 0.5 (+19.95 ± 0.05)
Scorch time (s)	70 ± 1	73 ± 1 (+4.3 ± 0.1)	72 ± 1 (+2.9 ± 0.1)
Air permeation (Barrer)	2.5 ± 0.1	1.26 ± 0.1 (−49.5 ± 2.1)	1.95 ± 0.03 (−21.7 ± 1.8)

## Data Availability

The data presented in this study are available on request from the corresponding author.
